# Metformin hydrochloride regulates glycolysis and inhibits PEDV replication by inhibition of PI3K-AKT signaling pathway

**DOI:** 10.1128/jvi.00147-26

**Published:** 2026-03-24

**Authors:** Xingcui Zhang, Yi Li, Jianbo Yuan, Qingyang Li, Xiaoru Hu, Ziyan Song, Zhiwei Sun, Yanwen Song, Yi Zhong, Guisong Liao, Jinman Ding, Shu Yang, Zhenhui Song

**Affiliations:** 1 Southwest University, College of Veterinary Medicine 26463, Chongqing, China; 2 Chuanshan Agricultural and Rural Affairs Bureau of Suining City, Suining, Sichuan Province, China; University of Kentucky College of Medicine, Lexington, Kentucky, USA

**Keywords:** PEDV, glycolysis, PI3K-AKT pathway, MH

## Abstract

Viruses rely on energy and biosynthetic materials of host cell from glucose metabolism to support their replication, and glucose plays a crucial role in viral infection. In this study, we found that porcine epidemic diarrhea virus (PEDV) infection significantly increased cellular glucose uptake and stimulated the production of the glycometabolite lactate. Exogenous supplementation of glucose or L-lactate confirmed that it significantly promoted PEDV proliferation, indicating that replication of PEDV was enhanced by regulating host glucose metabolism, particularly reprogramming glycolysis. Based on these findings, we explored the potential antiviral approach targeting the virus through regulating glycolytic processes. Metformin hydrochloride (MH) is a well-known hypoglycemic agent, which has shown notable anti-PEDV activity. After MH treatment, the transcriptome analysis showed the differential genes were mainly enriched in PI3K-AKT signaling pathway, and the expression levels of its downstream molecule GSK3B and MYC were significantly upregulated and downregulated, respectively. The gene expression related to glycolysis was also significantly inhibited. Further experiments showed that MH significantly inhibited the phosphorylation of AKT and its translocation to plasma membrane, while reducing the phosphorylation level of GSK3B. MH maintained GSK3B in a non-phosphorylated state by blocking the activation of the EGFR/PI3K/AKT/GSK3B pathway, mediated the degradation of c-MYC through phosphorylation, inhibited the glycolysis process, reduced the production of lactic acid, and finally exerted its antiviral effect. This study demonstrated that PEDV infection could induce glycolysis through metabolic reprogramming, thereby promoting viral replication; whereas, MH was able to effectively reverse this process, significantly inhibiting the virus-induced glycolysis pathway and exhibiting antiviral activity.

**IMPORTANCE:**

This study aims to elucidate the antiviral effects and molecular mechanisms of MH against PEDV. The results show that MH can inhibit the activation of the PI3K-AKT signaling pathway induced by PEDV infection, thereby suppressing the production of the glycolytic product L-lactic acid and ultimately resisting PEDV infection. This research provides new insights into the prevention and control of PEDV and offers scientific evidence for the application of MH in veterinary medicine.

## INTRODUCTION

PEDV belongs to the order *Nidoviridae*, family *Coronaviridae*, genus α *Coronavirus*, and is a single-stranded positive-sense RNA virus with a capsid (1). Its genome is about 28 kb in size and has a poly(A) tail at the 3′ end, a cap structure at the 5′ end, and seven open reading frames (ORF1a, ORF1b, and ORF2-6), encoding four structural proteins (Spike glycoprotein, Envelope protein, Membrane glycoprotein, Nucleocapsid protein), 16 non-structural proteins (nsp1-nsp16), and auxiliary protein ORF3, which coordinate the viral invasion, replication, and host immune evasion (2). PEDV primarily targets the villous epithelium of the porcine intestine, subsequently affecting the mucosa of the cecum, colon, and liver tissues. The virus causes severe diarrhea and high mortality in piglets by inducing the fusion and necrosis of intestinal epithelial cells.

Glycolysis converts glucose into pyruvate through a 10-step enzymatic process, providing the body with a large amount of ATP quickly. Enhanced glycolysis is common during disease, first observed in tumor cells, which require rapid energy to support quick, unlimited replication. Therefore, even in the presence of oxygen, tumor cells undergo glycolysis, a phenomenon known as the Warburg effect. Viruses lack their own material-energy metabolism and rely heavily on host cells to provide energy and material to complete their life cycle (3). Similar to tumor cells, viral infections also tend to glycolysis. For example, when murine norovirus (MNV) infects RAW264.7 cells, these cells show increased glycolysis, and researchers have noted that when glycolysis was inhibited, MNV replication was hindered (4).

The PI3K-AKT signaling pathway plays a vital role in regulating glucose metabolism, including PI3K, AKT, and GSK3Β. Activation begins when a ligand binds to the receptor tyrosine kinases, such as EGFR, and then PIP2 is formed into PIP3 via PI3K catalysis. This process recruits AKT to the plasma membrane and activates it through phosphorylation at Thr308 and Ser473. Once activated, AKT significantly influences glucose metabolism by phosphorylating targets such as GSK3B and GYS2 (5). It also inhibits the kinase activity of MYC and other oncogenic proteins by phosphorylation of GSK3B at Ser9, thus deregulating the regulation of c-MYC degradation (6). c-MYC promotes glucose uptake by directly inducing the expression of the glucose transporter GLUT1 and enhances the activity of key enzymes like hexokinase (HK2) and lactate dehydrogenase A (LDHA), thus supporting aerobic glycolysis (7, 8).

In this study, we found that MH showed strong antiviral activity both *in vivo* and *in vitro*. We demonstrated that MH could inhibit the activation of EGFR/PI3K/AKT/GSK3B and suppress the glycolysis process, thereby exerting antiviral effects. These findings are expected to stimulate the development of anti-PEDV drugs and novel methods targeting the glycolysis pathways.

## MATERIALS AND METHODS

### Cells and viruses

Porcine small intestinal epithelial cells (IPEC-J2) and African green monkey kidney cells (VERO-E6) were preserved by our group, and both cell lines were cultured in DMEM medium (Gibco, USA), supplemented with 10% fetal bovine serum (FBS, BI, Israel), and maintained at 37°C in the 5% CO₂ incubator. The PEDV-LJX strain was kindly donated by Liu Liang from Lanzhou Veterinary Research.

### Antibodies and reagents

The rabbit polyclonal antibody to PEDV N protein was prepared by our group, and the murine monoclonal antibody to PEDV N was kindly donated by Liu Liang from Lanzhou Veterinary Research. β-Actin Mouse Monoclonal Antibody, HRP-Goat Anti-Rabbit, HRP-Goat Anti-Mouse, EGFR Antibody, GSK3B Recombinant Antibody, GSK3B Monoclonal Antibody, Phospho-GSK3B (Ser9) Monoclonal Antibody, AKT Monoclonal Antibody, Phospho-AKT (Ser473) Mouse McAb, c-MYC Polyclonal Antibody, and EGFR Antibody were purchased from Proteintech (Wuhan). Phospho-EGF Receptor (Tyr1068) Antibody (CST Company), D-glucose anhydrous, L (+)-lactic acid, and MH were purchased from Solepol (Beijing, China). Amplex Red L-lactic acid assay kit and glucose assay kit (GOD/POD chromogenic method) were purchased from Beyotime (Shanghai, China).

### Cell viability assay

The well-grown IPEC-J2 cells were digested and diluted to 2 × 10^^5^/mL and then added to 96-well plate at a volume of 100 µL per well. When the cells reached approximately 90% confluence, 10 mM MH was prepared in DMEM basal medium at 11 concentrations: 5 µM, 10 µM, 20 µM, 40 µM, 60 µM, 80 µM, 100 µM, 120 µM, 160 µM, 200 µM, and 250 µM, incubated for 24 h. Then, CCK-8 reagent (Addison Biotechnology Co., Ltd., Jiangsu, China) was added to each well and incubated for 24 h. The results were measured using a Bio-MEM enzyme marker. The absorbance at 450 nm was recorded by the enzyme labeling instrument of the American Bio-Rad. Cell viability was calculated using the following formula: cell viability (%) = [OD (drug treatment) − OD (blank)]/[OD (control) − OD (blank)] × 100%.

### RT-qPCR

Total RNA was extracted from each cell sample using Trizol and then reverse-transcribed into cDNA with PrimeScript RT Master Mix (TaKaRa, Beijing, China). Specific primers for the PEDV N gene (GenBank No: MK252703.1) were designed, and the copy number of the PEDV N gene was quantified through absolute quantitative real-time PCR. The relationship between the cycle threshold (CT) values and the copy number was analyzed using the standard curve method in Bio-Rad CFX Manager software, and the copy number of the PEDV N gene was calculated. cDNA was amplified by qPCR using β-actin as an internal reference. The reaction mixture consisted of 5.0 μL SYBR PreMix Ex Taq II, 0.4 μL forward primer, 0.4 μL reverse primer, 1 μL cDNA, and 3.2 μL H_2_O. The protocol was as follows: 95°C for 30 s, then 40 cycles of 95°C for 5 s and 55°C for 30 s. Each sample was analyzed at least three times in duplicate.

### Western blot

Each sample was lysed on ice in 200 µL RAPI with 2 µL PMSF, and then the supernatant was collected quantified using the BCA Protein Concentration Assay Kit (Beyotime, Shanghai, China). The proteins were then subjected to denature quantitatively, separated by electrophoresis on SDS-PAGE gels (Beyotime, Shanghai, China), and then transferred to polyvinylidene fluoride (PVDF) membranes (Merck Millipore, USA). The membrane was blocked with the rapid blocking solution (Beyotime, Shanghai, China) for 10 min and then incubated with the appropriate primary antibodies at 4°C overnight. The next step was to incubate the membrane with the corresponding secondary antibody at room temperature for 1 h. Finally, the Western blot results were visualized using a BeyoECL Star (Beyotime, Shanghai, China) with the FX5 imaging system (VILBER, Israel).

### TCID_50_

VERO-E6 cells were seeded in the 96-well plates with the cell density 2 × 10^5^ cells/mL and 100 µL per well and then cultured the cells in a 37°C incubator. When cell confluence reached 90%, the PEDV-LJX virus solution was diluted serially 10-fold with DMEM basal medium, the DMEM basal medium group was used as a negative control, and six replicates were set for each group. After discarding the supernatant and washing with PBS, 100 µL of virus solution was added to each well and incubated at 37°C for 1 h. The cell culture fluid was then replaced with basal medium, and then, the cells were kept in the incubator for further cultivation. Then, the CPE of the cells was observed continuously. Within 7 days, 50% or more of the cells in the CPE were considered to be positive for lesions. The titer was calculated using the Reed-Muench method.

### Indirect immunofluorescence assay

IPEC-J2 cells were seeded at a density of 2 × 10⁴ cells per well into 24-well plates with coverslips. After treating the experimental groups, the cells were fixed with 4% paraformaldehyde for 25 min at room temperature, the fixative was removed, and cells were washed with PBS three times. The cells were permeabilized with 0.3% Triton X-100 (200 μL/well) for 10 min at 4°C and then blocked with 5% BSA diluted with PBST (200 μL/well) for 1 h at room temperature. Subsequently, the cells were incubated with PEDV N mouse anti-monoclonal antibody at 4°C overnight. After washing, Alexa Fluor 647-conjugated goat anti-mouse secondary antibody was applied and incubated in the dark at 37°C for 1 h. The cell nuclei were staining with DAPI in the dark at room temperature for 10 min. Fluorescence images were captured using a laser scanning confocal microscope (ZEISS, Germany). At least three independent replicates of each sample were set for each group.

### Transcriptome analysis and validation

To further investigate the molecular mechanism of MH inhibiting PEDV infection, differentially expressed genes were screened through transcriptome analysis for the PEDV group and the PEDV+MH group. Subsequently, RT-qPCR was used to verify the transcriptome results of the gene level.

### Animal experiment

Nine PEDV-negative piglets were purchased from a pig farm in Rongchang (Chongqing, China) and randomly divided into three groups: negative control (NC), PEDV-positive control (PEDV), and MH + PEDV, *n* = 3. Piglets were environmentally adapted and fed for 48 h before treatment, and each group was placed in an isolation room to prevent cross-infection. The pigs of blank group were orally administered with PBS in the morning; the PEDV infection group was given PEDV-LJX solution with a viral titer of 10^5^ TCID_50_/mL and 15 mL/kg; and the MH treatment group was orally administered with MH at 15 mg/kg body weight after oral administration of 15 mL/kg PEDV-LJX solution. The drug treatment lasted for 3 days. On the afternoon of the third day, all the piglets were euthanized; intestinal lesions were recorded through necropsy, and tissue samples of each intestinal segment were collected for subsequent experiments.

### Statistical analysis

All results were statistically analyzed using GraphPad Prism 9.0 software for analysis of variance (ANOVA) and *t*-tests. The data are presented as the standard error of the mean from three independent trials. *P* > 0.05 indicates no significant difference (ns); *P* < 0.05 (*) indicates a significant difference; *P* < 0.01 (**) and *P* < 0.001 (***) denote highly significant differences.

## RESULTS

### MH-inhibited PEDV infection *in vitro*

**Fig 1:**
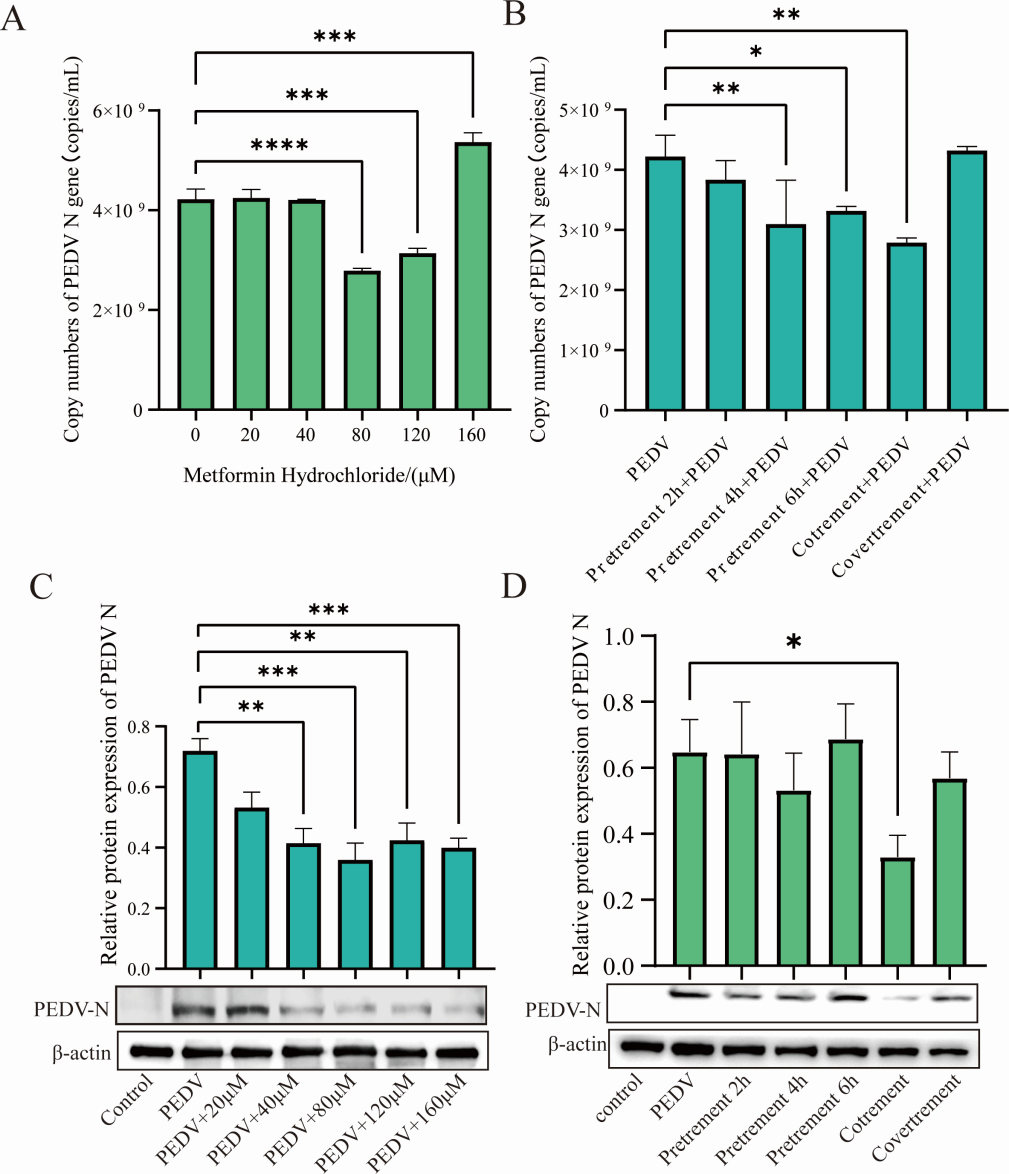
Determining the optimal concentration and treatment approach of MH for antiviral activity. (**A and C**) MH pre-treated the cells for 2 h with the concentrations of 20 µM, 40 µM, 80 µM, 120 µM, and 160 µM, respectively; the cells were infected with PEDV-LJX (MOI = 0.1); the RNA was extracted; and the levels of PEDV-N gene and protein expression were measured. (**B and D**) 80 µM MH was pretreated for 2 h, 4 h, and 6 h, respectively, and then, PEDV-LJX was added. Additionally, 80 µm MH was added for 1.5 h after PEDV-LJX inoculation as MH post-treatment. The gene and protein expression levels of PEDV-N were then detected.

Before the experiment of antiviral infection, the cytotoxicity of MH to IPEC-J2 cells was detected by CCK-8 assay, and the results showed that the cellular activity was higher than 50% at all MH concentrations less than 200 µM. The IPEC-J2 cells were pretreated and infected with PEDV at five different concentrations, and the cell viability was higher than 50%. The gene and protein levels of PEDV-N were detected by absolute RT-qPCR and Western blot to determine the optimal antiviral concentration. The Ct value of absolute RT-qPCR was substituted into the standard curve *Y* =−3.3823*X* + 56.47 to calculate the copy number of PEDV-N gene, and the results showed that 80 µM, 120 µM, and 160 µM concentrations of MH could play a good antiviral effect. Among them, MH with a concentration of 80 µM had the best antiviral effect (Fig. 1A and C), so the 80 µM concentration of MH was chosen for the subsequent experiments. To further determine the optimal treatment of MH, we set up several treatments of adding MH 2 h, 4 h, and 6 h in advance, adding it simultaneously with PEDV virus solution, and adding it after PEDV infection. The expression of PEDV N was detected by absolute RT-qPCR and Western blot. The results showed that the expression of the PEDV N protein was most significantly reduced in the co-treatment group (Fig. 1B and D). Therefore, the co-treatment was used for the subsequent experiment.

### MH showed antiviral effects at different times

**Fig 2:**
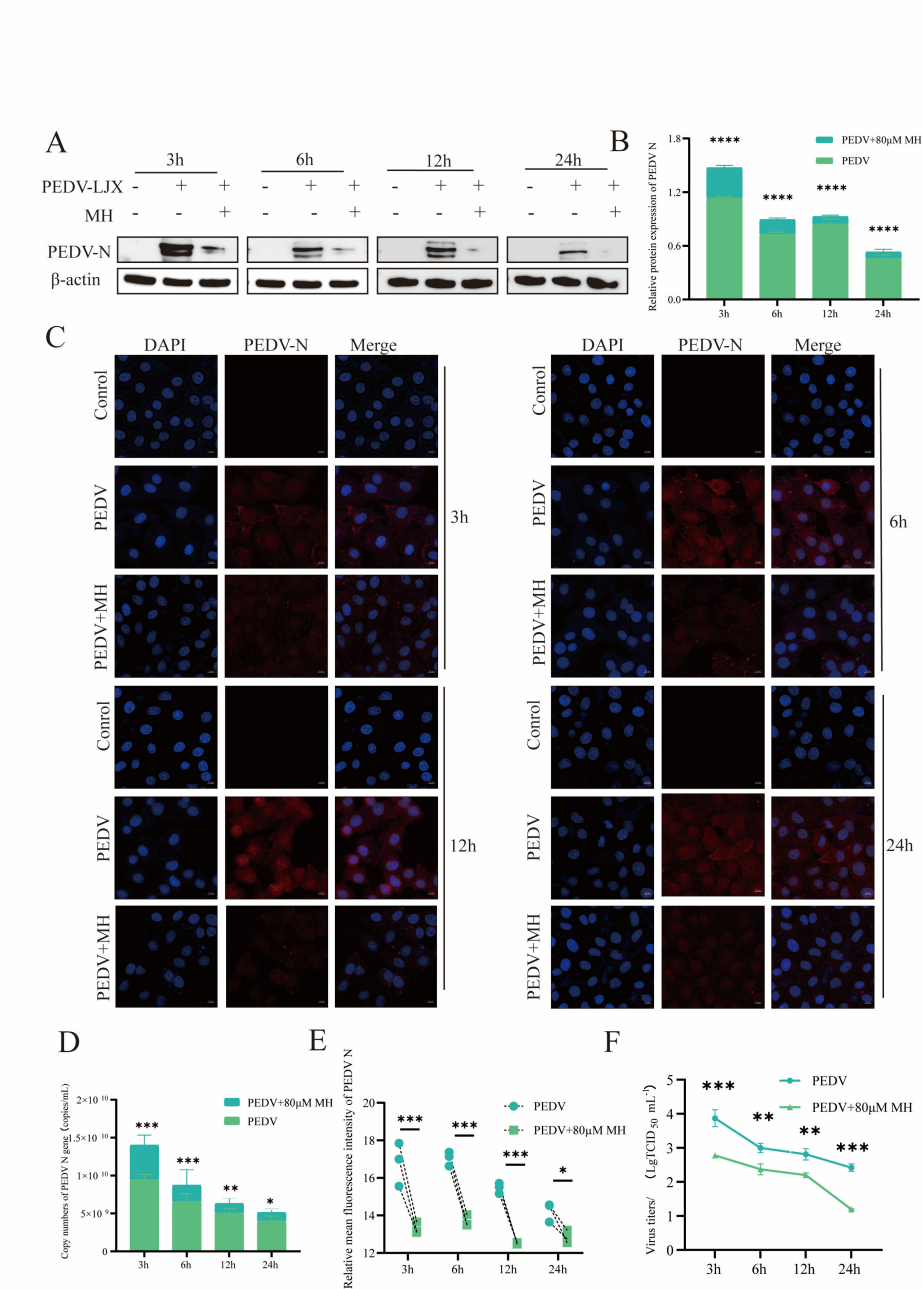
Antiviral effects of MH at different time points following viral infection. (**A**) Detection of the antiviral effects of MH at different time points by Western blot. (**B**) Gray scale analysis of PEDV-N protein expression. (**C**) Indirect immunofluorescence detection of the antiviral effect of MH at different times. (**D**) Absolute quantitative RT-qPCR analysis of PEDV-N mRNA levels. (**E**) Quantitative analysis of PEDV-N fluorescence. (**F**) Changes in PEDV viral titers at different times after treatment with MH.

To investigate the antiviral effect of MH at different time points treatment after viral infection, PEDV-N protein and gene levels were measured after MH treatment at 3 h, 6 h, 12 h, and 24 h. The results showed that gene and protein levels of PEDV-N were significantly reduced at 3 h, 6 h, 12 h, and 24 h after MH treatment (Fig. 2A, B and D). Additionally, immunofluorescence assay (IFA) was executed to detect the expression and relative position of PEDV-N. The analysis of the fluorescence distribution showed that the amount of PEDV was detected in the nucleus and cytoplasm of IPEC-J2 cells after PEDV infection. After MH treatment, the presence of PEDV N in these regions was significantly reduced. Fluorescence intensity analysis further confirmed that the intensity of PEDV N was extremely significantly reduced in the MH-treated group compared with the PEDV-infected group at 3 h, 6 h, and 12 h, and significantly reduced at 24 h (Fig. 2C and E). To determine whether MH acts by promoting the degradation of PEDV-N or by inhibiting the entire virus, we used TCID_50_ to detect titers after post-treatment with MH. The results showed that MH decreased the viral titers of PEDV at 3 h, 6 h, 12 h, and 24 h (Fig. 2F). These findings suggested that MH exhibits an effective antiviral activity at various stages of the viral life cycle.

### MH can inhibit the promoting effect of glucose on PEDV

**Fig 3:**
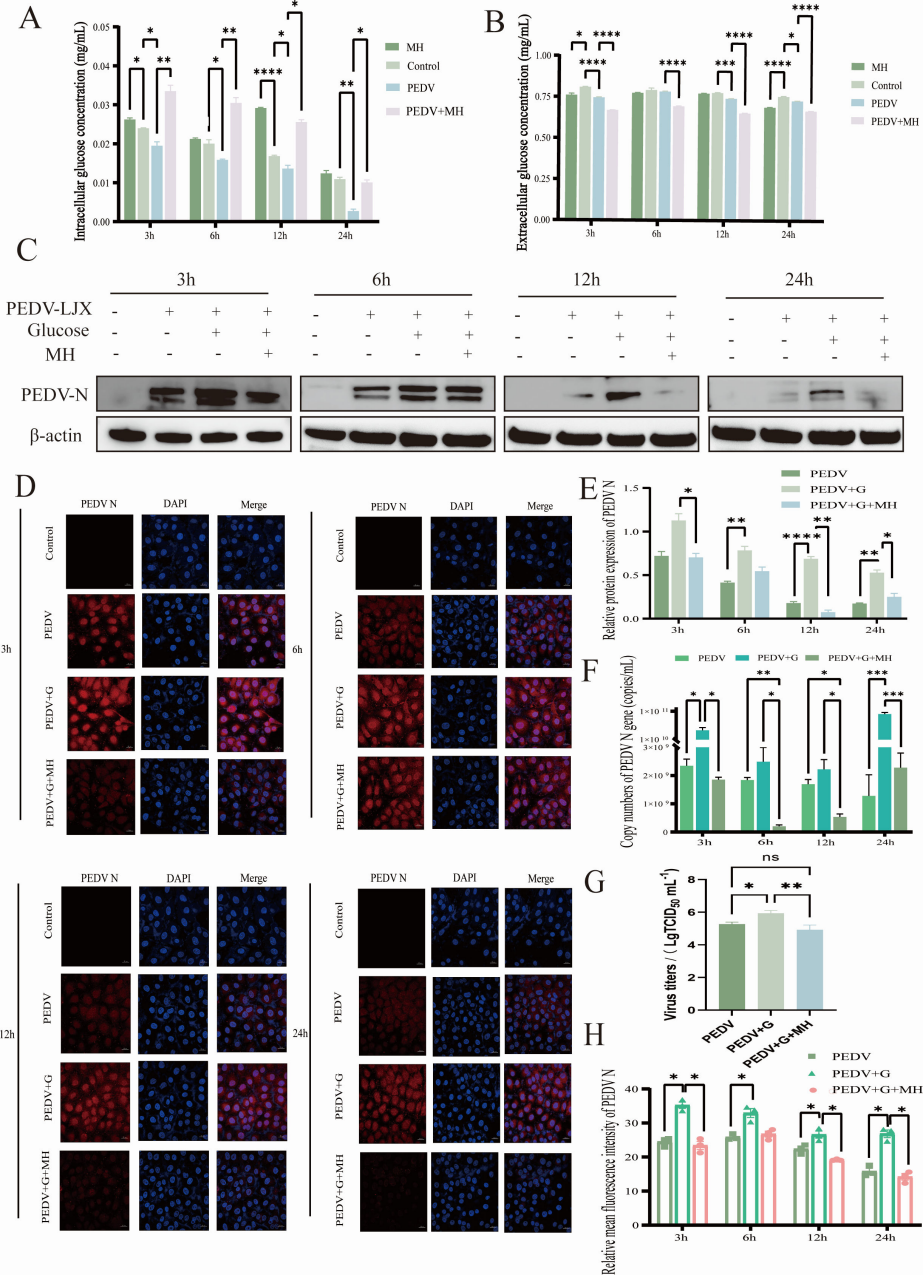
The antiviral function of MH is not mediated by regulation of glucose transport. (**A and B**) The change of glucose concentration in intracellular/extracellular. (**C**) Western blot detection of exogenous supplemental glucose as well as changes in PEDV-N protein levels under high glucose concentration conditions with MH. (**D**) Indirect immunofluorescence detection of fluorescence intensity as well as distribution of PEDV-N in different treatments. (**E**) Quantitative gray scale analysis of PEDV-N protein. (**F**) Absolute RT-qPCR detection of PEDV-N gene mRNA level. (**G**) Detection of the effect of exogenous glucose on the replication of PEDV by TCID_50_. (**H**) Quantitative analysis of PEDV-N fluorescence intensity.

Glucose is the core of the metabolism, and its level is closely related to cellular activity. We hypothesized that MH inhibits the effect of PEDV by regulating glucose transport. Intracellular and extracellular glucose levels were measured at different times following PEDV infection, MH treatment, or both simultaneous treatment with 80 μM MH. The results (Fig. 3A and B) showed that both MH treatment and PEDV infection could reduce extracellular glucose levels and increase intracellular glucose levels to vary degrees at different times. These data suggested that both PEDV infection and MH treatment promote glucose uptake, and the two have synergistic effects. Furthermore, we detected the level of PEDV-N protein after addition of exogenous glucose with or without MH treatment. RT-qPCR, Western blot, and IFA results all showed that exogenous glucose facilitated PEDV replication, while MH counteracted this promotion maintained significant antiviral effects (Fig. 3C through H). These findings suggest that high glucose levels support PEDV proliferation, and that MH can play an antiviral role by promoting glucose uptake even under high glucose conditions, but it does not inhibit the virus solely through regulating glucose uptake.

### MH regulates lactate levels to exert antiviral effects

**Fig 4:**
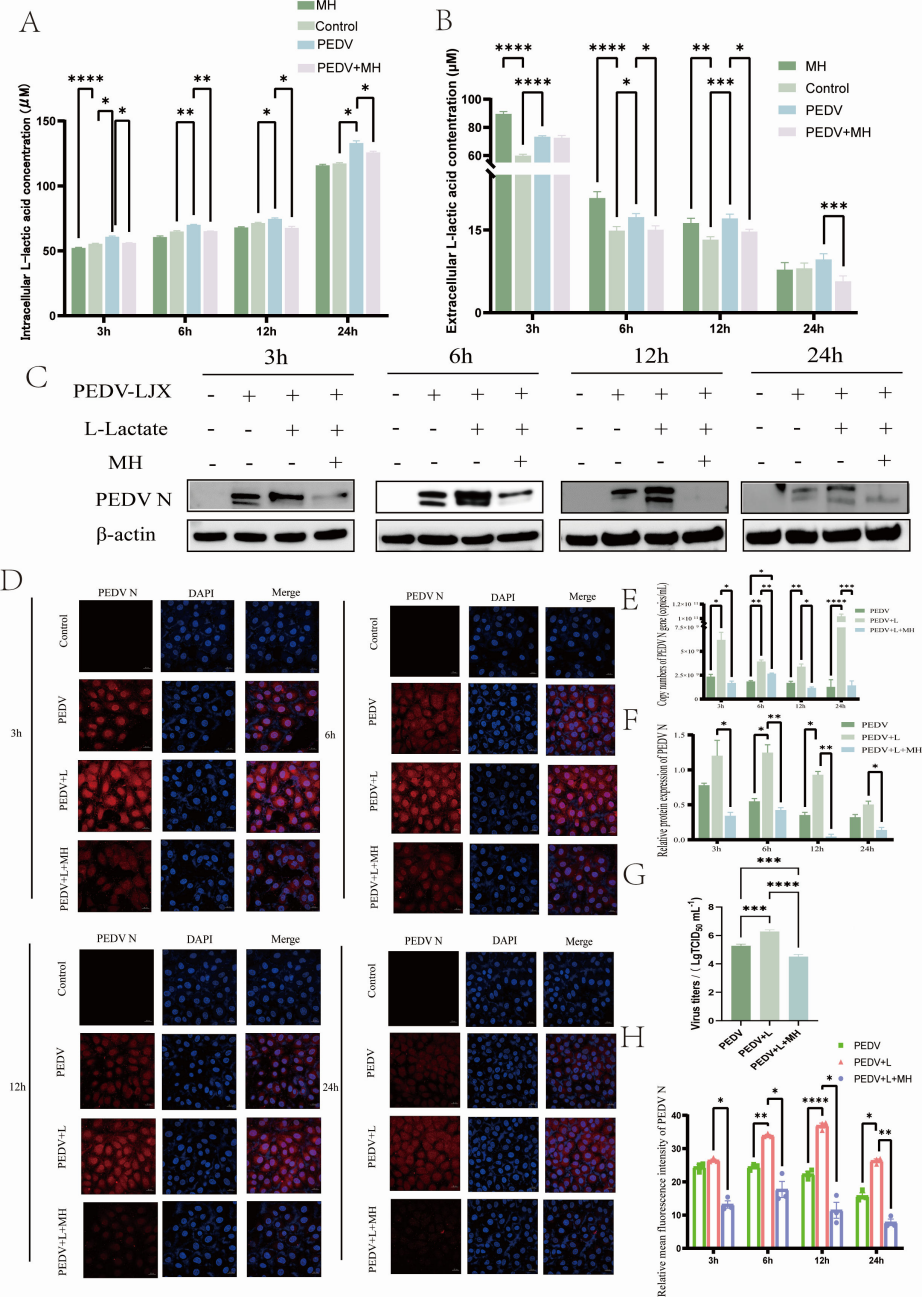
MH exerts antiviral effects by inhibition of L-lactate production and release. (**A**) Changes in the concentration of intracellular L-lactate. (**B**) Changes in the concentration of extracellular L-lactate. (**C**) Western blot was used to detect the changes of PEDV-N protein levels under the treatment of exogenous supplementation of L-lactate and high lactate concentration of MH. (**D**) Indirect immunofluorescence detection of the fluorescence intensity and distribution of PEDV-N in different treatments. (**E**) Absolute RT-qPCR detected the mRNA level of the PEDV-N gene. (**F**) Quantitative gray scale analysis of PEDV-N protein. (**G**) Detection of the effect of exogenous extracellular L-lactate on the replication of PEDV by TCID_50_. (**H**) Quantitative analysis of PEDV-N fluorescence intensity.

As shown in Fig. 4A and B, PEDV infection resulted in elevating the levels of intracellular and extracellular L-lactate, and MH was able to reduce these levels induced by PEDV. The abnormal increase of intracellular L-lactate caused by MH treatment may be due to the stimulation of glucose uptake by MH. The exogenous addition of L-lactate increased the expression of PEDV-N protein and genes, while MH countered this effect, inhibiting PEDV proliferation (Fig. 4C, E through G). In addition, we performed indirect immunofluorescence experiments to confirm that L-lactic promoted viral proliferation, while MH antagonized this process (Fig. 4D and H). These findings suggested that PEDV infection promotes the production and release of L-lactic. Moreover, under normal conditions, MH can promote the production of intracellular L-lactic, but its production and release are suppressed during PEDV infection. Overall, MH appears to exert its antiviral effects by suppressing the production and release of L-lactic.

### Correlation and validation of EGFR/PI3K/AKT/GSK3B signal axis related to transcriptomic predictions of PEDV infection

**Fig 5:**
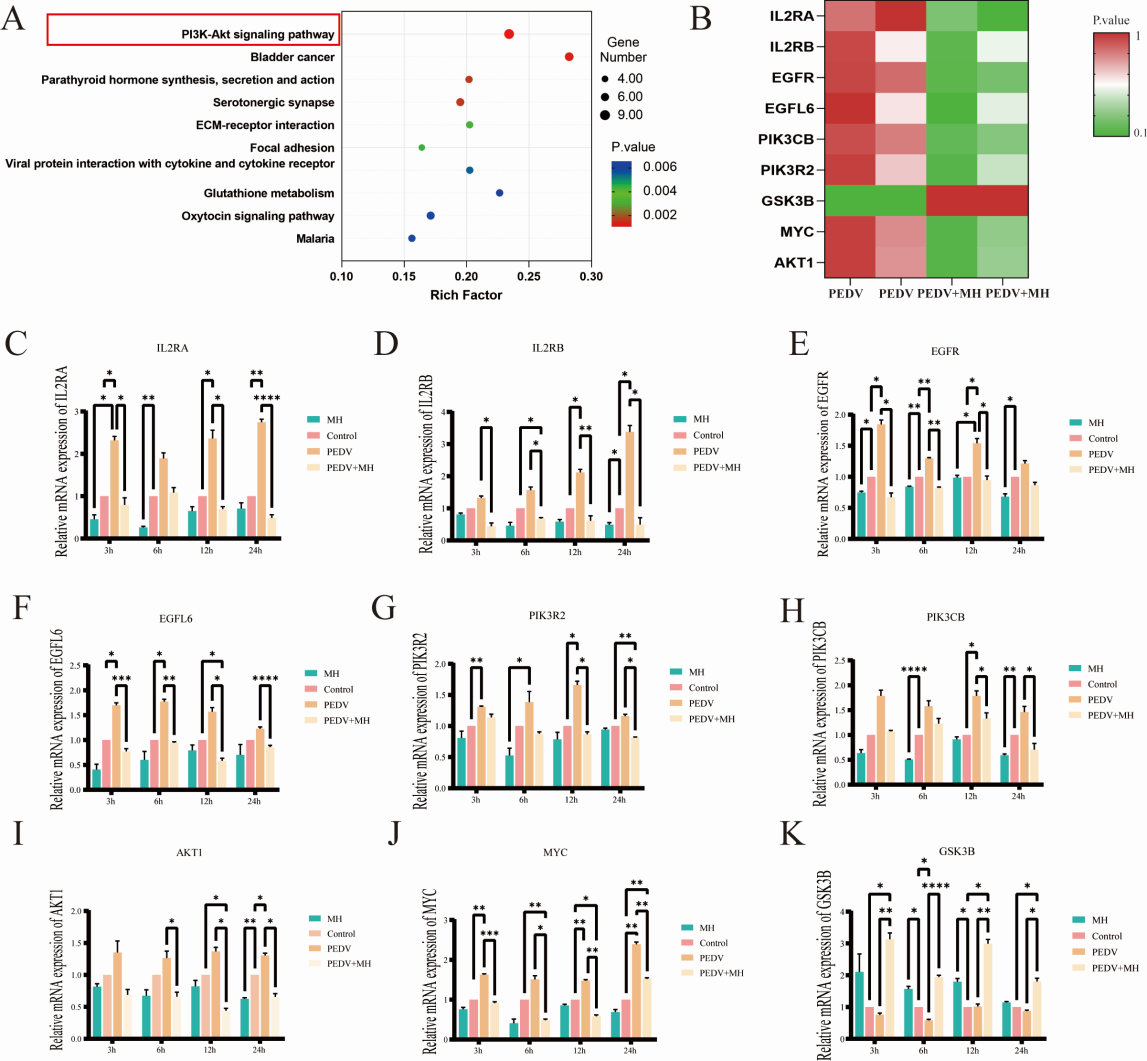
Transcriptome analysis and validation. (**A**) KEGG enrichment analysis. (**B**) Heat map showed significant differences in the expression of glycolysis-related genes between the PEDV-infected group and the MH + PEDV group. (**C–K**) Relative mRNA expression levels of IL2RA, IL2RB, EGFR, EGFL6, PIK3R2, PIK3CB, AKT1, MYC, and GSK3B genes.

Based on the results of transcriptomic, we counted the number of differentially expressed genes that were upregulated (log2FC >=1 & *P <* 0.05) and downregulated (log2FC <=-1 & *P <* 0.05). Compared to the PEDV-infected group, 161 genes were significantly upregulated and 117 genes were significantly downregulated in the MH + PEDV group. KEGG enrichment analysis revealed that these differentially expressed genes were significantly enriched in the PI3K-AKT signaling pathway (Fig. 5A). Further heat map analysis of key genes in the pathway showed that MH treatment caused downregulation of IL2RA, IL2RB, EGFR, EGFL6, PIK3CB, PIK3R2, MYC, and AKT1 and upregulation of GSK3B (Fig. 5B).

To ensure the reliability and reproducibility of the transcriptomic data, the expression levels of the relevant genes were validated using RT-qPCR. The results showed that the expression levels of IL2RA, IL2RB, EGFR, EGFL6, PIK3CB, PIK3R2, MYC, and AKT1 genes were significantly higher in the PEDV-infected group compared with those of the blank control group, while the expression level of GSK3B was significantly lower. In the MH treatment group, the expression levels of IL2RA, IL2RB, EGFR, PIK3CB, and AKT1 were significantly decreased, and the expression level of GSK3B was significantly increased. Compared with the PEDV infection group, the MH + PEDV group showed significant decreased expression levels of IL2RA, IL2RB, EGFR, EGFL6, PIK3CB, PIK3R2, MYC, and AKT1 and significantly increased expression level of GSK3B (Fig. 5C through K). These findings were consistent with the transcriptome results.

### MH inhibits EGFR/PI3K phosphorylation activation induced by PEDV

**Fig 6:**
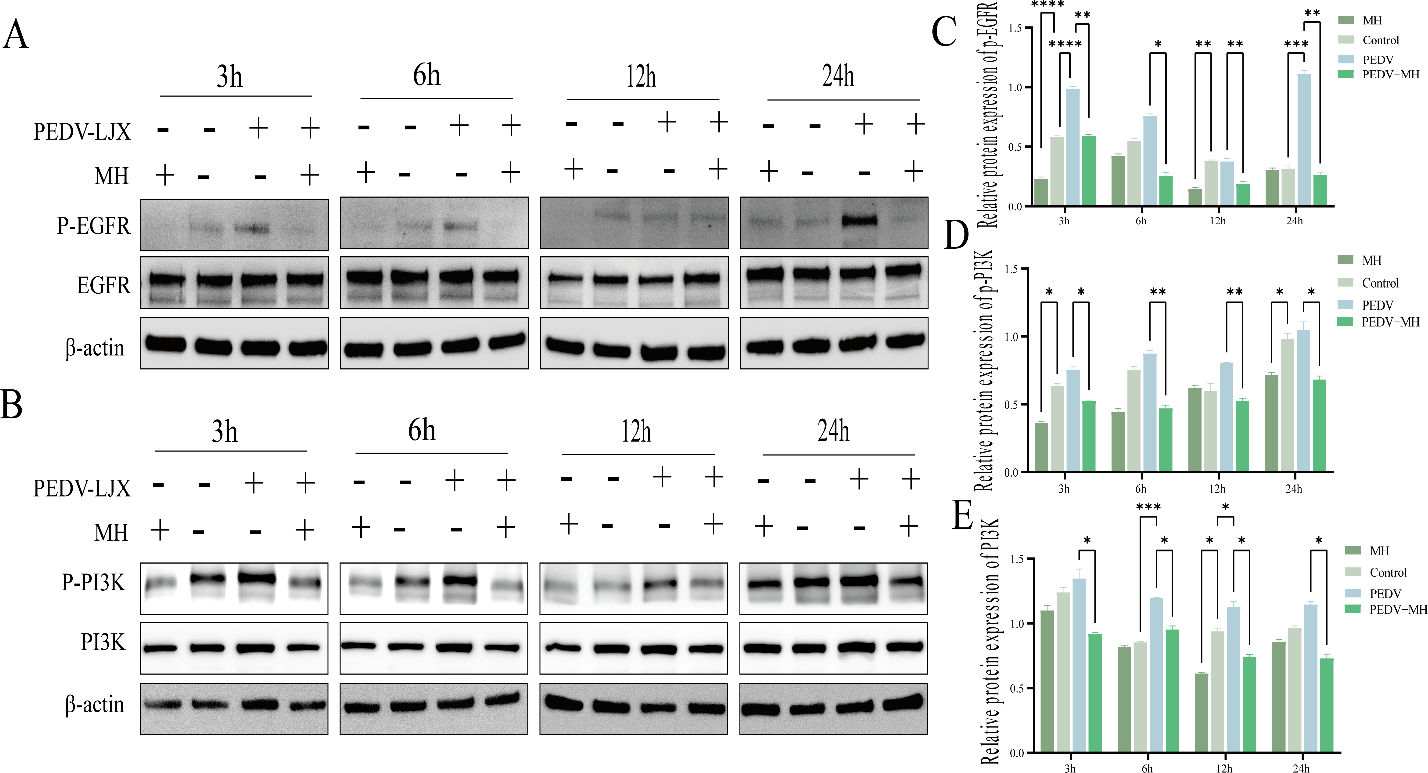
MH inhibits PEDV-induced activation of EGFR-PI3K phosphorylation. (**A**) Western blot detection of EGFR and p-EGFR protein expression levels after MH treatment, PEDV infection, and PEDV infection with simultaneous MH treatment. (**B**) Western blot detection of PI3K and p-PI3K protein expression levels following MH treatment, PEDV infection, and PEDV infection combined with MH treatment. (**C**) Gray-scale analysis of p-EGFR protein. (**D and E**) Gray-scale analysis of p-PI3K/PI3K proteins.

Phosphorylation of EGFR is the initial signal for PI3K-AKT activation. When cells are stimulated by external factors, EGFR on the cell surface is activated through phosphorylation. p-EGFR can regulate cellular stress through a variety of pathways, including the regulation of glycolysis by phosphorylating PI3K. Following treatment with MH, PEDV infection, or simultaneous treatment with MH and PEDV, we detected EGFR and p-EGFR levels using Western blot. The results (Fig. 6A and C) showed that PEDV infection increased EGFR phosphorylation, while MH inhibited its phosphorylation. As MH inhibited EGFR phosphorylation, it may also inhibit the activation of the downstream PI3K phosphorylation. Therefore, we assessed the protein levels of p-PI3K and PI3K under the same conditions. The results were consistent with our expectations: PEDV infection promoted PI3K phosphorylation, while MH effectively inhibited PI3K phosphorylation (Fig. 6B, D and E). These findings suggest that PEDV infection enhances the phosphorylation of downstream PI3K by promoting EGFR phosphorylation, which, in turn, activates PI3K. Conversely, MH appears to block the phosphorylation of EGFR and PI3K proteins.

### MH inhibits AKT phosphorylation induced by PEDV

**Fig 7:**
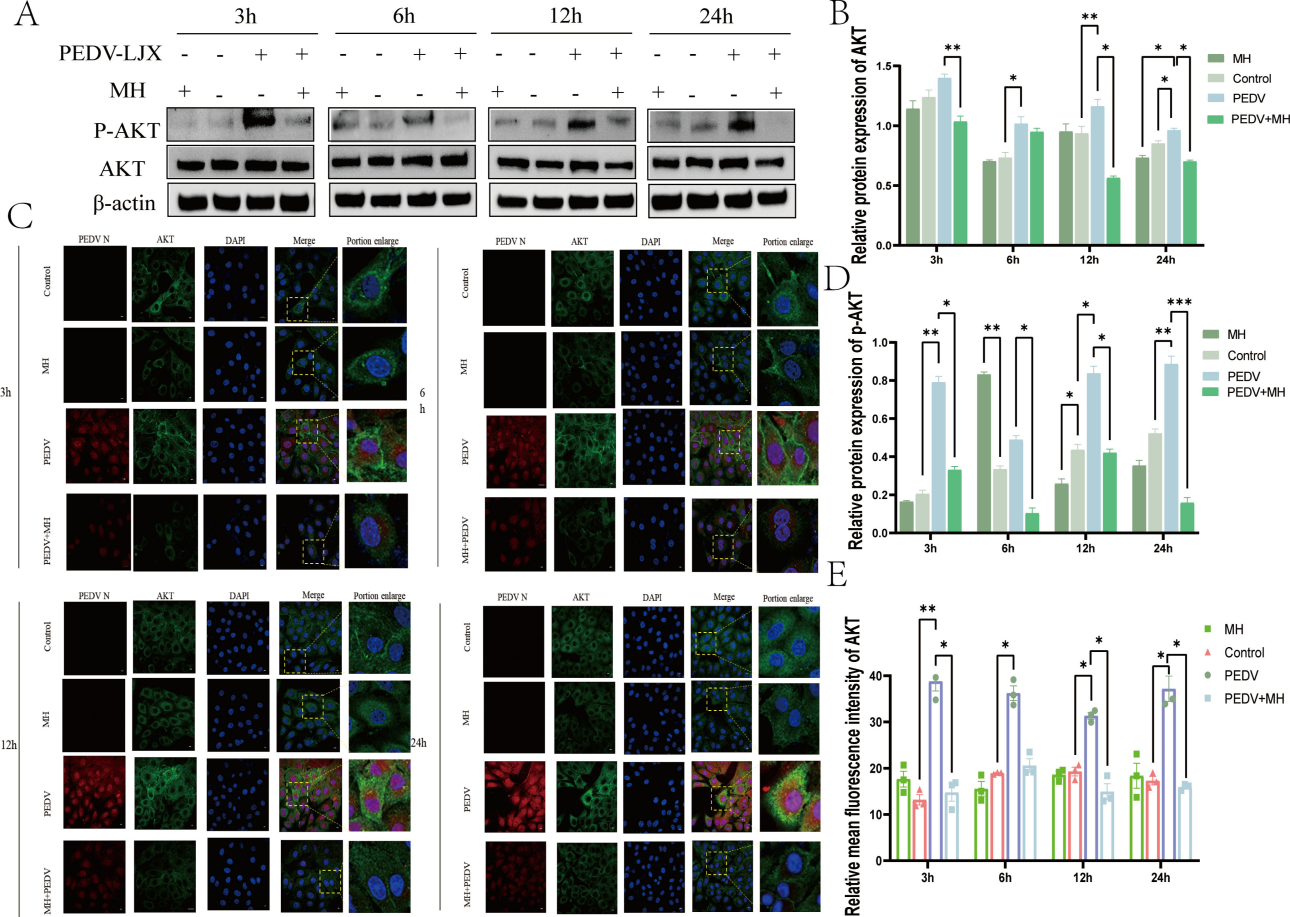
PEDV infection promotes AKT expression and phosphorylation activation, and MH counteracts AKT activation. (**A**) Western blot detection of the effects of MH treatment, PEDV infection, and PEDV infection combined with MH on AKT and p-AKT levels. (**B**) Gray scale analysis of AKT protein. (**C**) Immunofluorescence detection of AKT and PEDV-N subcellular localization and expression levels. (**D**) Gray scale analysis of p-AKT protein. (**E**) Fluorescence intensity analysis of AKT.

AKT is a key regulatory factor downstream of PI3K, and the activation of PI3K phosphorylation can promote the translocation of AKT to the plasma membrane and its phosphorylation. As shown in Fig. 7A, B and D, we found that PEDV infection promoted AKT phosphorylation and led to an increased expression level of AKT. However, MH treatment inhibited this process. Furthermore, we examined the expression level changes of AKT subcellular localization and PEDV-N levels using IFA (Fig. 7C), the results were consistent with that of the western blot: PEDV infection increased the expression of AKT, while MH suppressed this phenomenon by inhibiting PEDV infection (Fig. 7E), and the changes trend of PEDV-N was consistent with that of AKT. The key process of AKT phosphorylation includes the transfer to the plasma membrane. During the late stage of PEDV infection (24 hpi), the level of AKT decreased, and the protein was distributed around the plasma membrane, indicating that PEDV infection promoted the transfer and phosphorylation of AKT at the membrane. Conversely, MH treatment reduced AKT levels and caused its distribution to be uniform in the cytoplasm. Overall, these results suggest that PEDV infection induces AKT expression and promotes its phosphorylation, while MH exerts antiviral effects by preventing the translocation of AKT to the cell membrane and reducing the expression and phosphorylation levels of PI3K.

### MH inhibition of PEDV-induced GSK3B phosphorylation can promote c-MYC degradation

**Fig 8:**
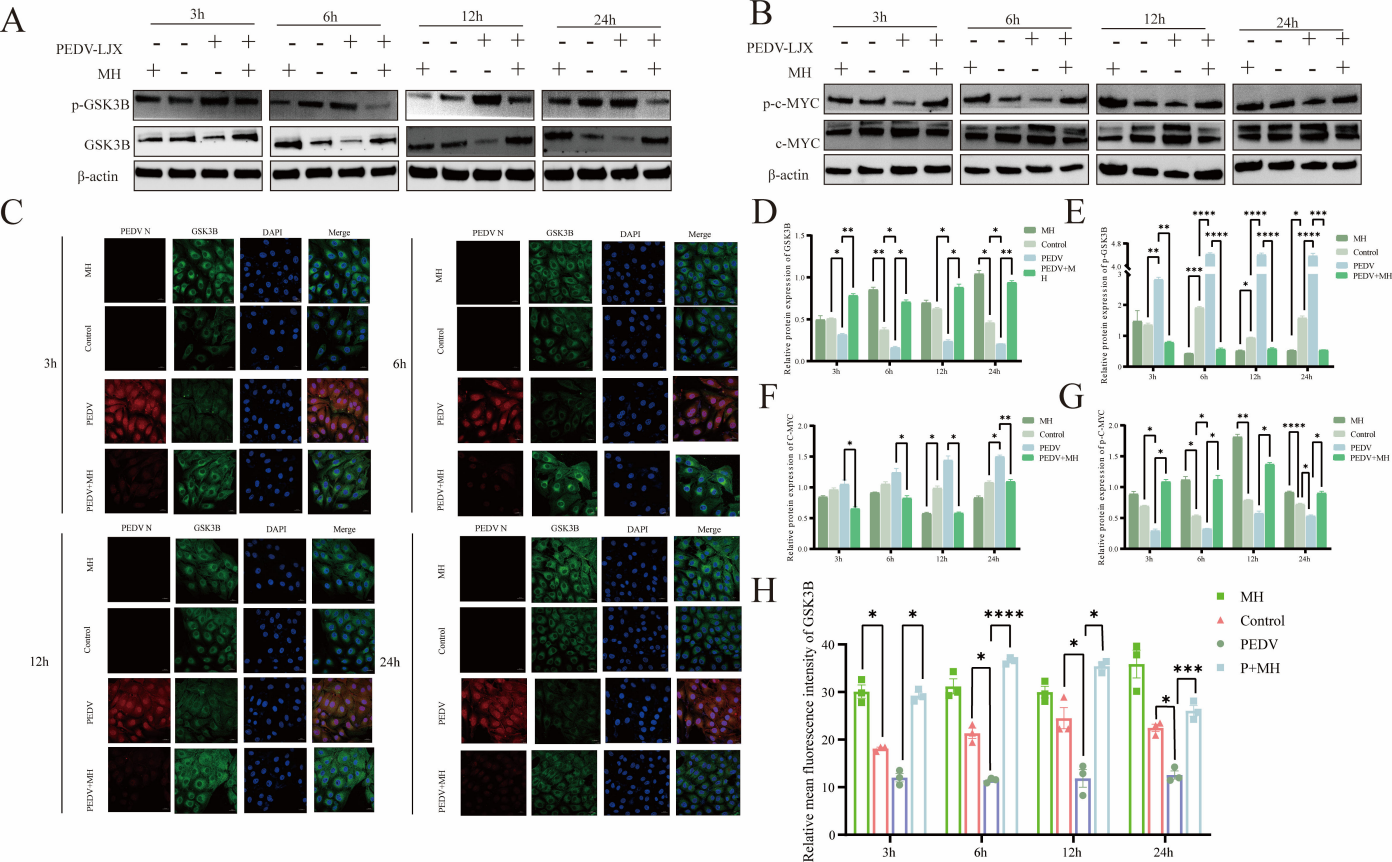
MH regulates the phosphorylation of GSK3B and c-MYC induced by PEDV infection. (**A**) Western blot detection of the effects of MH treatment, PEDV infection, and PEDV infection combined with MH on GSK3B and p-GSK3B levels. (**B**) Western blot detection of the effects of MH treatment, PEDV infection, and PEDV infection combined with MH on c-MYC and p-c-MYC levels. (**C**) Immunofluorescence detection of GSK3B and PEDV-N subcellular localization and expression levels. (**D and E**) Gray scale analysis of GSK3B/p-GSSK3B proteins. (**F and G**) Gray scale analysis of c-MYC/ p-c-MYC proteins. (**H**) Fluorescence intensity analysis of GSK3B.

GSK3B and c-MYC are key genes downstream of the PI3K-AKT signal pathway involved in regulating glycolysis. Activated AKT prevents the degradation of the c-MYC protein by phosphorylating GSK3B, allowing c-MYC to enter the nucleus, regulating the expression of glycolysis-related enzymes, and promoting the glycolytic process. As shown in Fig. 8A, B and D through G, PEDV infection increased the phosphorylation of GSK3B and significantly reduced the phosphorylation of the downstream signal molecule c-MYC. Conversely, MH played an opposite role, which inhibited the phosphorylation of GSK3B under both normal and PEDV-infected conditions, and increased the phosphorylation level of c-MYC, promoting the degradation of c-MYC. In addition, we used indirect immunofluorescence to detect the changes in the level of GSK3B, a key kinase regulating c-MYC phosphorylation, and its correlation with the level of PEDV-N protein (Fig. 8C and H). The results were consistent with the previous findings: PEDV infection enhanced GSK3B phosphorylation and decreased GSK3B levels, while MH inhibited GSK3B phosphorylation under both normal and infected conditions. This maintained the function of GSK3B and promoted the phosphorylation-dependent degradation of c-MYC. We also observed a negative correlation between the levels of GSK3B and PEDV-N, and the inhibition of GSK3B phosphorylation by MH appears to be the central of its antiviral effect.

### MH inhibits the expression of glycolysis-related genes

**Fig 9:**
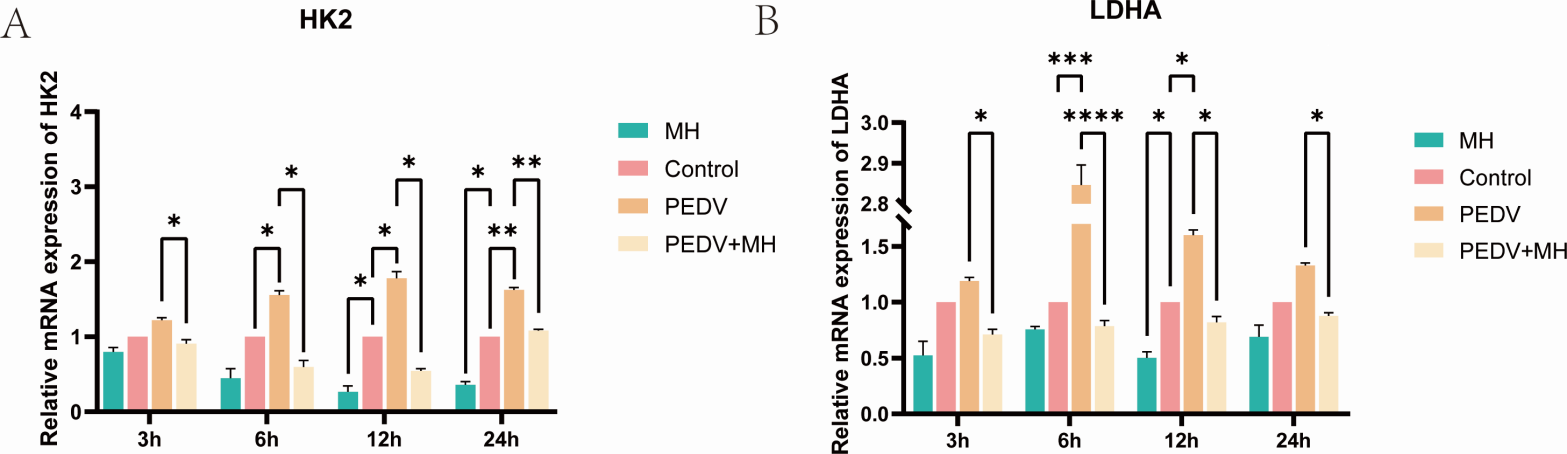
The effect of PEDV and MH on the expression of the glycolytic genes. (**A and B**) The mRNA expression levels of HK2 and LDHA genes were detected by RT-qPCR.

It was demonstrated that c-MYC enhances the transcription of glycolysis-related proteins HK2 and LDHA by entering the nucleus and binding to the promoter. Therefore, we used relative RT-qPCR to detect the transcription of glycolysis-related genes. The results showed that the expression levels of HK2 and LDHA genes were significantly higher in the PEDV-infected group than in the control group and significantly decreased in the group treated with MH. Conversely, the expression levels of HK2 and LDHA genes were significantly lower in the MH + PEDV group compared to those of the PEDV-infected group (Fig. 9A and B).

### MH inhibits PEDV infection in piglets

**Fig 10:**
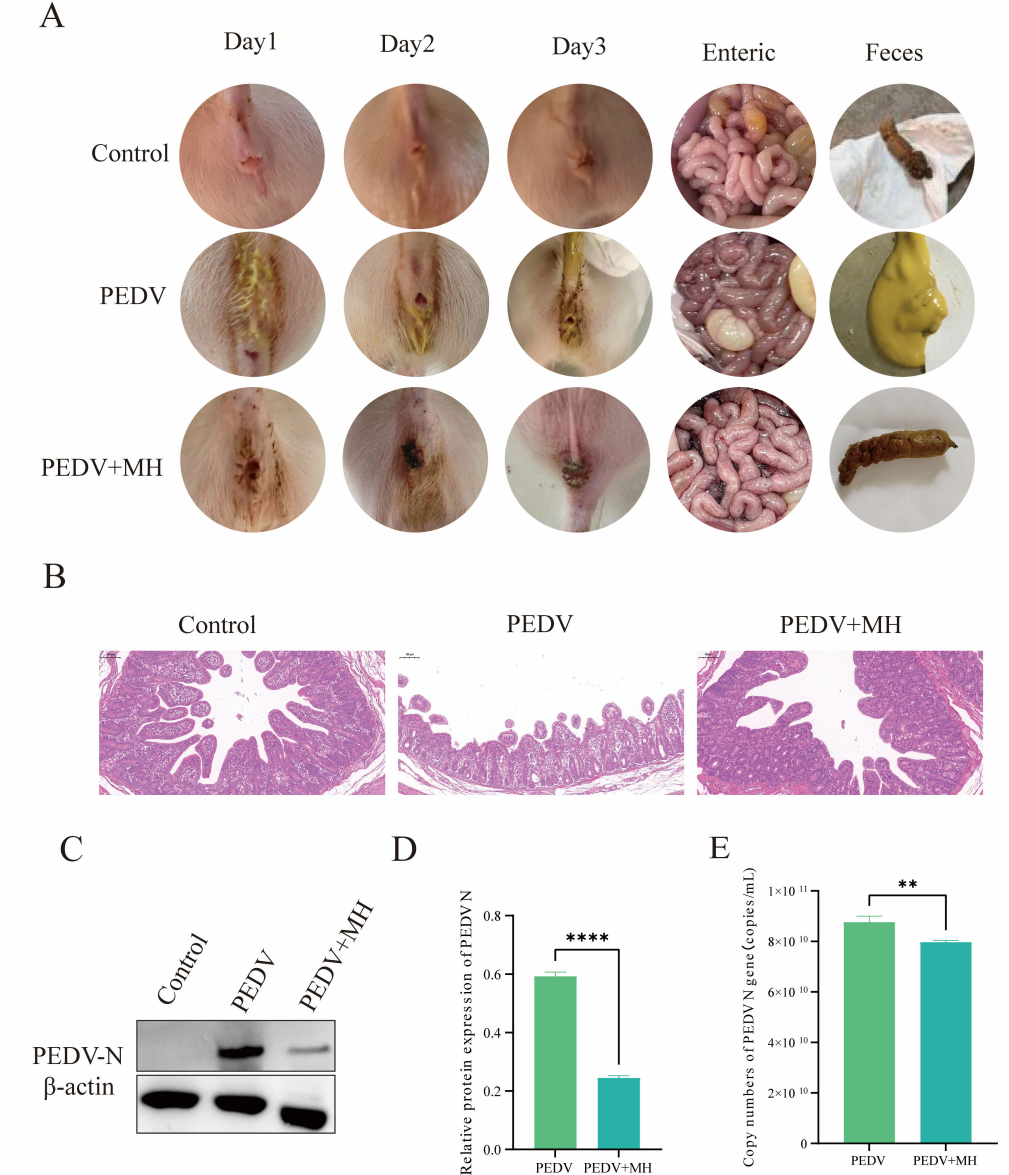
Antiviral effect of MH *in vivo*. (**A**) Feces and intestinal changes in the PEDV-infected group and MH-treated group. (**B**) Changes of PEDV-N levels detected by Western blot under the MH hydrochloride treatment. (**C**) PEDV-N protein gray scale analysis. (**D**) Relative protein expression of PEDV N. (**E**) Absolute RT-qPCR detection of PEDV-N gene expression levels.

*In vitro*, we found that MH had efficient antiviral effects and questioned whether it could exert the same effects *in vivo*. To further investigate this, we selected Rongchang pigs under 7-day old to establish an infection model and treated them with MH. After 3 days of consecutive treatment, the piglets infected with PEDV showed obvious clinical symptoms, with watery feces, and obvious intestinal lesions were found at necropsy. However, postmortem examination showed that after MH treatment, the symptoms of the suckling were significantly alleviated, the feces became drier, and the intestinal lesions were relieved (Fig. 10A). The pathological histological results showed that in the PEDV infection group, the small intestinal villi atrophy and shorten, but in the PEDV + MH group, it was found that the damage degree of the small intestinal villi was significantly relieved (Fig. 10B). We detected the PEDV-N protein by Western blot and RT-qPCR, and the results (Fig. 10C through E) showed that PEDV-N protein and gene levels were significantly lowered after MH treatment. This demonstrated that MH could also exert a strong antiviral effect *in vivo*.

## DISCUSSION

In recent years, the widespread occurrence of PEDV, the diversity of viral serotypes, and the complexity of mutations have posed significant challenges to the prevention and control of the virus (9). These factors also threaten the pig industry due to their high infectiousness and lethality. In China, the G1 strain was predominant until 2010, both live attenuated vaccines and inactivated vaccines have provided effective immunological protection (10). However, with the spread and mutation of the virus, traditional vaccines can no longer achieve the desired protective effect. Therefore, the development of novel antiviral drugs has become a new focus in the prevention and treatment of PEDV.

The ability of viruses to acquire material and energy from host cells is essential for viral proliferation. To obtain these resources more efficiently, many viruses have evolved to induce a shift in the host metabolic phenotype towards aerobic glycolysis, where the cell preferentially generates energy through the glycolytic pathway even in an oxygen-rich environment; this is known as the Warburg effect. Numerous viruses can hijack glycolysis to support their proliferation and spread. For example, HPV infection causes increased glycolysis, elevated lactate production and lipid accumulation in the host; this HPV-induced enhancement of glycolysis promotes the onset and progression of cervical cancer and is key to HPV carcinogenesis (11). Certain viral proteins can directly regulate the expression of glycolysis-related gene in the host—for instance, the adenovirus-encoded E 4 ORF1 protein activates the transcription of glycolytic genes by interacting with MYC transcription factors (12, 13). A similar mechanism exists in coronaviruses, where SARS-CoV-2 non-structural protein Nsp6 interacts with the host MGA/MAX complex, remodeling the host metabolic network and increasing glycolytic flux for viral replication (14). However, some viruses adopt the opposite regulatory strategies, such as the hepatitis B virus (HBV), which limits glycolysis and promotes glutamine metabolism by inhibiting HK2 activity to meet the needs of replication (15). Metabolic reprogramming is a significant feature of the pathogenesis process of PEDV infection. PEDV can reprogram a variety of metabolic pathways in the host, downregulating heterogeneous nuclear ribonucleoprotein A 3 (HNRNPA3) via mir-218-5p, activating sterol regulatory element-binding protein 1 (SREBF1) to promote lipid synthesis-providing raw materials for the formation of the viral envelope; simultaneously, it inhibits AMPK-mediated lipophagy, reduces fatty acid β-oxidation, and maintains accumulation of intracellular lipid droplets. In terms of energy metabolism, PEDV induces high-glucose glycolysis in host cells and establishes a “Warburg effect” by upregulating the activity of pyruvate kinase M 2 (PKM2) to supply ATP and nucleic acid precursors for viral proliferation. PEDV infection promotes glucose uptake and lactate production and release. Lactate accumulation may further enhance glycolysis feedback signaling (e.g., through the HIF-1α signaling pathway) to provide more materials for viral replication. PEDV may take advantage of this metabolic. The key glycolytic genes such as HK2 and LDHA significantly increased, and transcriptomic data further showed the upregulation of glycolysis-related genes. Our experimental results support the conclusion that PEDV infection promotes glycolysis.

Targeted modulation of virus-hijacked glucose metabolism has provided new insights for the development of antiviral drugs. For example, the inhibition of glycolysis by 2-deoxy-D-glucose (2-DG) severely reduced the spread of IAV in a dose-dependent manner (16). In a study of SARS-CoV-2 infection, researchers found that melatonin treatment restored the redox equilibrium in COVID-19 patients by inhibiting the Warburg effect (17). The STING proteins significantly reduced the viral titers in HSV-1 infection models by inhibiting the mitochondrial localization and enzymatic activity through binding to HK2 (18).

MH plays an important role not only in the treatment of type 2 diabetes, but also in recent years, more and more evidence has shown that it has application potential in antiviral, antitumor, and antiaging. In HPV viral infection, viral E6 and E7 oncoproteins are crucial for the proliferation of HPV-positive cancer cells. Studies have shown that MH significantly inhibits the progression of HPV-positive cancer cells by targeting and suppressing the expression of the E6/E7 cancer proteins, thereby preventing their development (19). In the novel coronavirus SARS-CoV-2, MH, as a hypoglycemic agent, reduces expression of entry factors and SARS-CoV-2 infection in cultured hepatocytes under pathological glucose conditions (20). However, its effects on PEDV have not been reported. We performed transcriptomic analysis on samples from the PEDV-infected and MH-treated groups. The data showed that MH inhibited the expression of glycolysis-related genes and reduced the production and secretion of L-lactate. These findings suggest that MH counteracts PEDV-induced enhancement of glycolysis. The upregulation of glycolysis by viruses is a double-edged sword: on the one hand, glycolytic intermediates such as 3-phosphoglycerate can promote the secretion of IL-1β through activating NLRP3 inflammasome, thus creating a pro-inflammatory environment conducive to viral proliferation; on the other hand, excessive glycolysis can lead to lactic acid accumulation, which can potentially result in cellular acidification, thereby inhibiting viral replication. This paradoxical phenomenon has been demonstrated in dengue virus (DENV) infection (21). To explore how increased glycolysis influences PEDV infection, we exogenously added glucose to IPEC cells post-infection and observed a significant increase in viral load compared to the control group without glucose addition. This indicates that the increased glycolytic substrates promote PEDV replication. To evaluate the effect of MH on the virus, we treated cells both *in vitro* and *in vivo* and measured the levels of PEDV-N. The results demonstrated that MH has efficient antiviral activity in both settings. In summary, MH exerts its antiviral role by antagonizing virus-induced glycolysis. Previous studies have reported that MH affects the internalize stage of the virus lifecycle (22). In our subsequent experiments, we will further investigate which stage of the PEDV lifecycle is impacted by the MH molecule.

The PI3K-AKT signaling pathway is a crucial pathway for regulating glucose metabolism, and many viruses reprogram host metabolism by hijacking this pathway. For example, human cytomegalovirus (HCMV) activates the PI3K-AKT-mTOR signaling cascade, significantly upregulating the expression of HK2 and LDHA, leading to a large amount of pyruvate being converted to lactate instead of entering the tricarboxylic acid cycle (23). This metabolic shift not only quickly generates ATP to support viral assembly but also accumulates key precursors such as glucose 6-phosphate to provide materials for nucleic acid synthesis during viral genome replication (24). On the other hand, the alphavirus nonstructural protein Nsp3 mediates phosphorylation activation of the PI3K-AKT signaling pathway through its YXXM motif, which, in turn, upregulates the expression of the glucose transporter protein GLUT1 and promotes cellular glucose uptake (25). A key step in the activation of the PI3K signal pathway is the activation of AKT, namely its phosphorylation. The activated AKT regulates glucose metabolism, uptake, and conversion by phosphorylating multiple downstream targets such as GSK3B and GYS2 (5). The activation of GYS2 promotes the conversion of glucose in the blood to glycogen (26). GSK3β regulates glucose metabolism, and in its unphosphorylated state, GSK3Β functions to degrade MYC; however, phosphorylation by activated AKT abolishes this ability, leading to the accumulation of c-MYC, which promotes metabolic reprogramming and enhances glycolysis (27). In this study, we observed that PEDV infection promoted the phosphorylation of EGFR, AKT, and GSK3Β and significantly increased the levels of c-MYC through Western blot, RT-qPCR, and IFA. c-MYC enhanced the expression of glycolysis-related genes and shifted the metabolism of IPEC-J2 cells toward glycolysis. After MH treatment, AKT phosphorylation, membrane localization, and the phosphorylation levels of EGFR and GSK3Β were reduced, and the level of c-MYC also significantly decreased. These results suggest that MH exerts an antiviral effect by inhibiting the activation of the PI3K-AKT-GSK3Β signal pathway.

**Fig 11:**
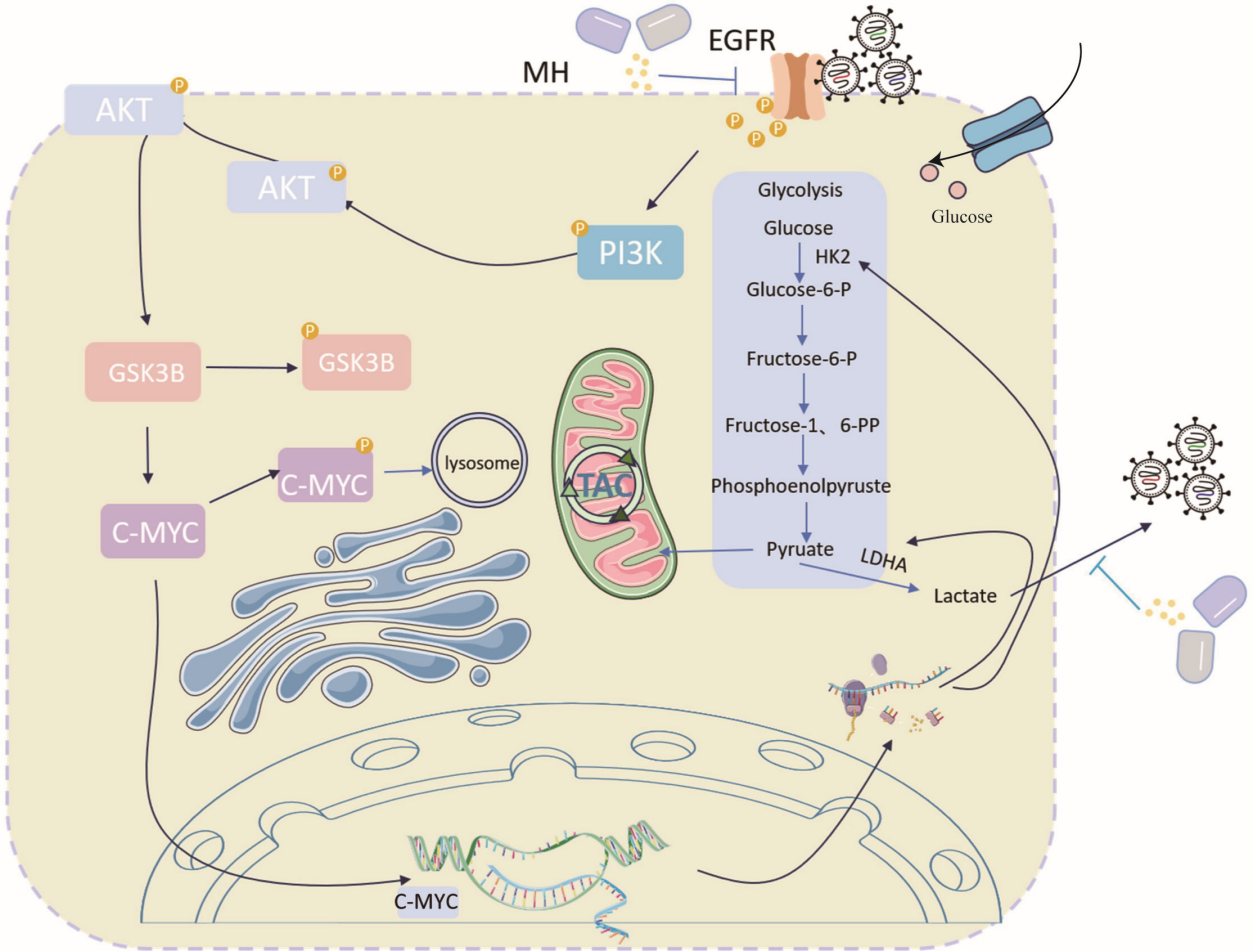
The mechanism of MH inhibiting PEDV replication by regulating glycolysis through EGFR/PI3K/AKT/GSK3B signaling axis.

In conclusion, this study demonstrates that PEDV infection promotes viral proliferation by hijacking glycolysis, while MH plays an antiviral role both *in vivo* and *in vitro* as follows: MH inhibits the activation of the PI3K-AKT signaling pathway, inhibits the phosphorylation of GSK3Β, and accelerates the degradation of c-MYC, thereby reducing the expression of key glycolytic proteins HK2 and LDHA (Fig. 11). This inhibits PEDV-induced glycolysis, decreases production and secretion of lactic acid, and enhances antiviral activity. The dynamic balance of lactate metabolism is particularly important in viral infection: lactate dehydrogenase-mediated pyruvate-lactate conversion not only maintains the NAD+ regeneration in the cytoplasm to ensure the continuous function of glycolytic but also indirectly affects the activation of viral genes by modulating chromatin accessibility through histone lactate modification (28, 29). The observed positive correlation between lactate levels and viral load suggests that PEDV may utilize epigenetic remodeling induced by lactate accumulation to promote its replication.

Lactic acid accumulation can reduce the pH value both extracellularly and intracellularly, forming an acidic microenvironment. Many viruses, including coronaviruses, are more likely to complete the fusion of the envelope with the host cell membrane under slightly lower pH conditions, thus promoting the entry of the virus into the cell. Moreover, lactic acid accumulation can also stabilize viral proteins: an acidic environment may help maintain the conformational stability of certain viral proteins (such as S protein), thus enhance the viral infectivity. In addition, lactic acid is known to have immunomodulatory effects, such as inhibiting the production and signaling of interferon (IFN), suppressing function of cytotoxic T cells and NK cells, and promoting the activity of immunosuppressive cells such as regulatory T cells (Treg) or M2 type macrophages. These effects could potentially weaken the host’s ability to clear PEDV, thereby indirectly promoting viral replication (30). Lactic acid metabolism may interact with mitochondrial function, affecting reactive oxygen (ROS) levels. Moderate ROS may promote PEDV replication by activating signaling pathways that viral replication, such as the MAPK/ERK pathway. PEDV is known to induce endoplasmic reticulum stress and utilize the autophagic pathway to promote its replication, and lactate accumulation may exacerbate endoplasmic reticulum stress or regulate the autophagic process, thus being “hijacked” the virus to facilitate its replication. PEDV primarily infects intestinal epithelial cells, and the intestine itself is an environment that is relatively hypoxic and prone to lactate production. Lactic acid produced by the intestinal microbiota may also affect the local microenvironment, potentially regulating the PEDV infection. However, these hypotheses warrant further investigation.

## Data Availability

The data generated during and/or analyzed during the current study are included in the article.
